# Griffithsin-mediated inhibition of cellular entry of hemorrhagic fever viruses and insights into its mechanisms

**DOI:** 10.1128/jvi.00372-26

**Published:** 2026-04-27

**Authors:** Takeshi Saito, Wakako Furuyama, Devinda S. Muthusinghe, Yannick Munyeku-Bazitama, Takanari Hattori, Mami Okabe, Takeshi Yokoyama, Yoshikazu Tanaka, Ryuichi Sakai, Yasuteru Sakurai, Miako Sakaguchi, Rika Tsukagoshi, Noelia S. Coronado Barrios, Thomas Tipih, Kyle Rosenke, Andrea Marzi, Manabu Igarashi, Shuzo Urata, Junki Maruyama, Asuka Nanbo, Ayato Takada

**Affiliations:** 1 Department of Pathology, University of Texas Medical Branch 12338, Galveston, Texas, USA; 2 National Research Center for the Control and Prevention of Infectious Diseases, Nagasaki University 12961, Nagasaki, Japan; 3 Division of Global Epidemiology, International Institute for Zoonosis Control, Hokkaido University 12810, Sapporo, Japan; 4 Institut National de Recherche Biomédicale 309878, Kinshasa, Democratic Republic of the Congo; 5 Département de Biologie Médicale, Faculté de Médecine, Université de Kinshasa 482996 , Kinshasa, Democratic Republic of the Congo; 6 Faculty of Medicine, University of Kikwit 601651, Kikwit, Democratic Republic of the Congo; 7 Graduate School of Life Sciences, Tohoku University 89255, Sendai, Japan; 8 Hokkaido University Faculty and Graduate School of Fisheries Sciences 204958, Hakodate, Japan; 9 Institute of Tropical Medicine, Nagasaki University, Nagasaki, Japan; 10 Laboratory of Virology, Rocky Mountain Laboratories, Division of Intramural Research, National Institute of Allergy and Infectious Diseases, National Institutes of Health 828462 , Hamilton, Montana, USA; 11 International Collaboration Unit, International Institute for Zoonosis Control, Hokkaido University 12810, Sapporo, Japan; 12 One Health Research Center, Hokkaido University 12810, Sapporo, Japan; 13 Department of Disease Control, School of Veterinary Medicine, University of Zambia 247512 , Lusaka, Zambia; The Ohio State University, Columbus, Ohio, USA

**Keywords:** Griffithsin, hemorrhagic fever virus, Ebola virus, Marburg virus, Lassa virus, Lujo virus, Crimean-Congo hemorrhagic fever virus, entry, antiviral

## Abstract

Griffithsin (GRFT), a lectin derived from the red algae *Griffithsia*, is known as an antiviral agent that binds to high-mannose glycans on viral envelope glycoproteins (GPs). In this study, we evaluated the antiviral activity of GRFT against Ebola virus (EBOV), Marburg virus (MARV), Lassa virus (LASV), Lujo virus (LUJV), and Crimean-Congo hemorrhagic fever virus (CCHFV), all of which cause severe and often fatal hemorrhagic fevers in humans. GRFT effectively blocked the entry of these viruses into cells, with stronger inhibition observed against LASV, LUJV, and CCHFV than against EBOV and MARV. This inhibitory effect depended on the lectin activity of GRFT, as its mutant lacking glycan-binding function completely lost the ability to inhibit the entry of these viruses into cells. We found that GRFT induced aggregation of virus-like particles (VLPs) derived from EBOV and MARV, whereas this activity was not observed with LASV- and LUJV-derived VLPs. Most of the escape mutants of LASV and LUJV GPs exhibited amino acid substitutions that likely disrupted GRFT binding by removing glycosylation sites at positions 79 and 73, respectively. Interestingly, other substitutions close to these positions, although unrelated to glycosylation, also contributed to reduced inhibitory activity of GRFT, suggesting a unique recognition mode in which both sugar chains and adjacent amino acid residues constitute the binding site structure for GRFT. Overall, our results provide insights into mechanisms underlying the antiviral effects of GRFT and highlight lectin-related activities as a key feature for broad-spectrum antiviral agents targeting highly glycosylated envelope GPs.

**IMPORTANCE:**

Emerging and re-emerging infectious diseases, including viral hemorrhagic fevers, pose a major global health threat due to their potential for widespread outbreaks. Currently, treatment options for such diseases are limited and often ineffective against newly emerging viruses. Here, we demonstrate that Griffithsin (GRFT), a naturally derived lectin from red algae, inhibits the entry of multiple hemorrhagic fever viruses into host cells. By blocking key processes of the viral entry mediated by envelope glycoproteins, lectins such as GRFT can exhibit broad antiviral activity that could potentially overcome the limitations of existing treatments against emerging viruses. Our findings emphasize that targeting sugar chains and adjacent structures on viral glycoproteins with GRFT could provide a therapeutic strategy against diverse viral species. Such an approach is particularly valuable for newly emerging viruses for which specific countermeasures have not yet been established.

## INTRODUCTION

Viral hemorrhagic fevers are severe, often lethal diseases, predominantly caused by viruses belonging to certain virus families, such as *Filoviridae*, *Arenaviridae*, and *Nairoviridae* (1). Filoviruses, such as Ebola virus (EBOV) and Marburg virus (MARV), are notable for their high case fatality rates of up to 90% and their potential to cause outbreaks with devastating public health and socioeconomic impacts (2). Arenaviruses, such as Lassa virus (LASV) and Lujo virus (LUJV), and nairoviruses, such as Crimean-Congo hemorrhagic fever virus (CCHFV), are also known to cause hemorrhagic fever in humans. Transmission of these viruses occurs through contact with infected reservoir animals or arthropod vectors, although human-to-human transmission via body fluids is also common in EBOV, MARV, and LASV infection (1). EBOV and MARV have caused frequent outbreaks in sub-Saharan Africa since their first discovery, including those recently reported from previously unaffected countries (2, 3). LASV is endemic in West Africa and annually causes outbreaks with case-fatality rates among hospitalized patients ranging from 15% to 70% (4, 5), while CCHFV has a wide geographic distribution encompassing Africa, the Middle East, Asia, and parts of Europe, aligning with the habitat range of its primary vector, *Hyalomma* ticks (6). LUJV is a relatively newly discovered pathogen with only a few documented cases, and information on this virus is quite limited (7).

Currently, there are few available therapeutics for viral hemorrhagic fevers. For EBOV, although two monoclonal antibody drugs (Inmazeb [REGN-EB3] and Ebanga [mAb114]) have been approved by regulatory authorities in the USA and Europe (8), challenges remain with regard to versatility. These therapeutic antibodies are effective against EBOV but not other human-pathogenic filoviruses, such as Sudan virus and Bundibugyo virus, both of which have caused sporadic outbreaks in African countries (2). For LASV and CCHFV, ribavirin remains the primary treatment option despite limited data on human efficacy (9, 10), and several drug candidates, including monoclonal antibodies and small molecule compounds like favipiravir, are still in various stages of development (11–14).

Lectins are a diverse group of carbohydrate-binding proteins widely distributed in nature, from plants to mammals, and are characterized by their ability to selectively recognize specific glycans (15, 16). Bacterial- and plant-derived lectins have been extensively investigated as antiviral drug candidates (16, 17). Griffithsin (GRFT), derived from the red algae *Griffithsia*, is a lectin that exhibits a strong binding affinity for high-mannose glycans, which are prominently displayed on many viral envelope glycoproteins (GPs) (18, 19). It has been shown that this unique binding property enables GRFT to effectively inhibit the cellular entry of various enveloped viruses, including HIV-1, SARS-CoV, MERS-CoV, SARS-CoV-2, Nipah virus, Andes virus, and EBOV (18–24). By targeting glycan structures at functionally important sites on viral GPs, GRFT prevents viral attachment to and/or membrane fusion with host cells, interrupting critical steps in the viral lifecycle. Thus, GRFT, which shows broad-spectrum antiviral activity, is expected to be a promising candidate for therapeutic development as a novel antiviral agent for combating newly emerging and re-emerging viruses. Here, we show that GRFT inhibits the entry of representative hemorrhagic fever viruses (i.e., EBOV, MARV, LASV, LUJV, and CCHFV) into target cells and provide insights into the mechanisms underlying the inhibitory activity.

## RESULTS

### GRFT inhibits cellular entry of hemorrhagic fever viruses

**Fig 1:**
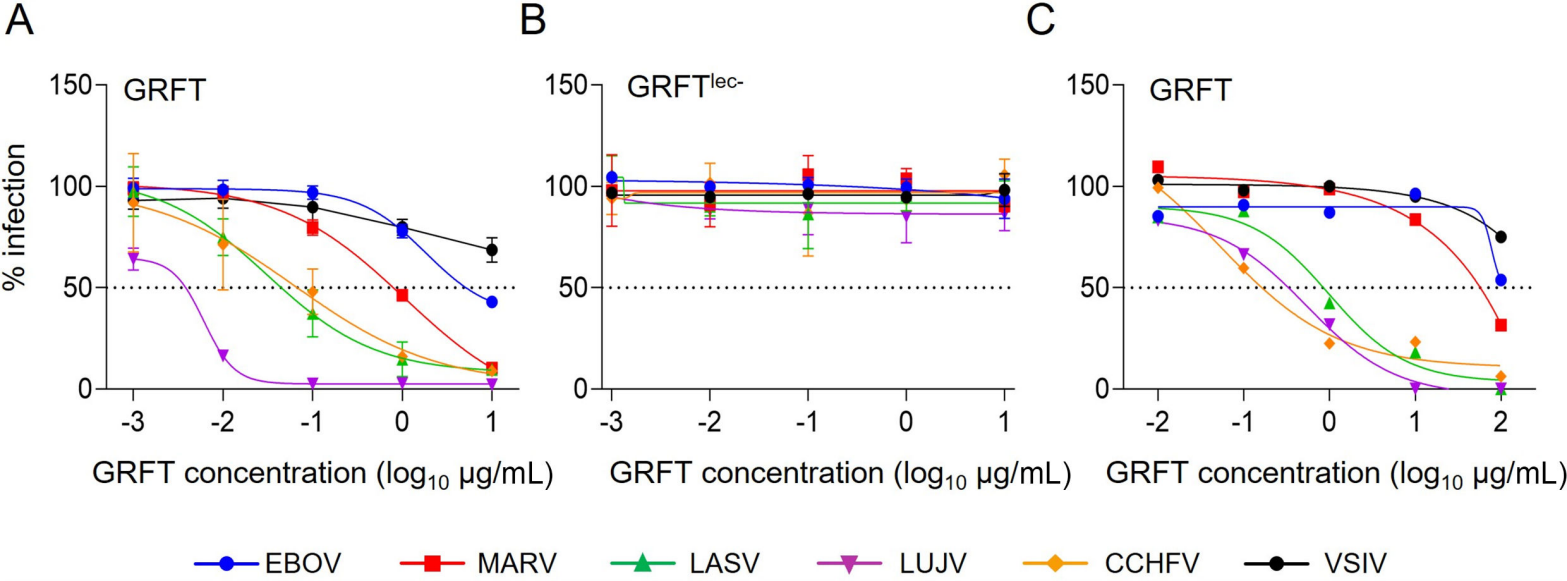
Inhibitory activity of GRFT against hemorrhagic fever viruses. Pseudotyped VSIVs were mixed with the indicated concentrations of wild-type GRFT (**A**) or its mutant GRFT^lec−^ (**B**) and inoculated onto Vero E6 cells, and the numbers of GFP-expressing cells were counted. Each experiment was performed in triplicate, and averages and standard deviations are shown. Infectious hemorrhagic fever viruses were mixed with the indicated concentrations of wild-type GRFT (**C**) and inoculated onto Vero E6 cells, and the numbers of plaques were counted. Relative infectivity was calculated compared to the negative control (i.e., without GRFT) for each virus.

Vero E6 cells were infected with replication-incompetent vesicular stomatitis Indiana virus (VSIV) pseudotyped with the envelope GP of EBOV, MARV, LASV, LUJV, CCHFV, or VSIV (VSVΔG*EBOV, VSVΔG*MARV, VSVΔG*LASV, VSVΔG*LUJV, VSVΔG*CCHFV, or VSVΔG*VSIV, respectively) in the presence or absence of GRFT (Fig. 1A; Table S1). GRFT inhibited infection with VSVΔG*EBOV and VSVΔG*MARV with 50% inhibitory concentrations (IC_50_) of 5.11 and 0.79 µg/mL, respectively, whereas its inhibitory effect against VSVΔG*VSIV was limited. Interestingly, the antiviral activities of GRFT against VSVΔG*LASV, VSVΔG*LUJV, and VSVΔG*CCHFV were much greater than those against the other viruses tested, with IC_50_ values of approximately 0.044, 0.0038, and 0.067 µg/mL, respectively. These results indicated that GRFT inhibited GP-mediated entry of the tested hemorrhagic fever viruses into cells. To confirm the requirement of lectin activity for the observed antiviral potential of GRFT, we generated a mutant GRFT^lec−^ with three amino acid substitutions (D30A, D70A, and D112A) previously shown to abolish the glycan-binding function of GRFT (25) and tested its ability to inhibit infection with the above-mentioned pseudotyped viruses (Fig. 1B). As expected, none of the viruses were significantly inhibited by GRFT^lec−^ even at the highest concentration tested (10 µg/mL). Then, the inhibitory activity of GRFT was evaluated in plaque-reduction tests using actual isolates of EBOV, MARV, LASV, LUJV, CCHFV, and VSIV (Fig. 1C; Table S2). We confirmed that GRFT inhibited plaque formation of these hemorrhagic fever viruses, although the IC_50_ values were higher than those against the pseudotyped viruses (Tables S1 and S2), most likely due to the differences in assay procedures and the density of viral surface GPs between authentic and pseudotyped virus particles (26–28). Consistent with the inhibitory activities against the pseudotyped viruses, LASV, LUJV, and CCHFV were more efficiently inhibited by GRFT than EBOV and MARV.

### GRFT induces aggregation of Ebola and Marburg virus-like particles

**Fig 2:**
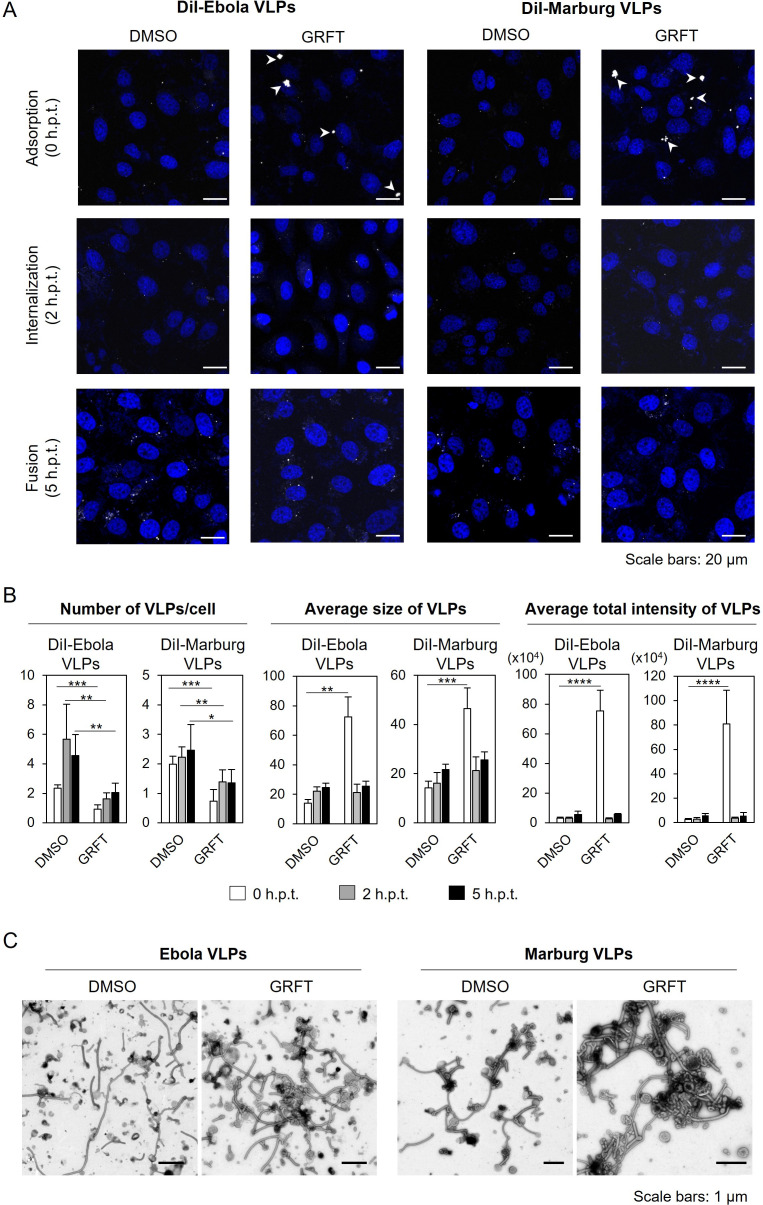
Fluorescent and ultrastructural observation of VLPs derived from filoviruses. (**A and B**) Visualization of Ebola and Marburg VLPs. DMSO- or GRFT-treated DiI-labeled Ebola and Marburg VLPs were adsorbed into Vero E6 cells and incubated for 30 min at room temperature. After adsorption, the cells were incubated at 37°C. At 1.5 post-temperature shift (h.p.t.), the cells were treated with trypsin. DiI signals on the cell surface and in the cytoplasm were monitored at 0, 2, and 5  h.p.t. using confocal laser scanning microscopy (**A**). The nuclei were stained with 1 μg/mL Hoechst 33342. Scale bars represent 20 μm. The numbers of DiI signals in the cells, average size, and average total fluorescence intensity of the DiI signals were quantified (**B**). The data represent the means ± standard deviations of three independent experiments. Statistical analysis was performed using Student’s *t*-test (*P  <  0.05, **P  <  0.01, ***P  <  0.001). (**C**) Electron microscopy of Ebola and Marburg VLPs. Purified Ebola or Marburg VLPs were incubated with DMSO or GRFT for 30 min at 37°C, followed by negative staining. Scale bars: 1 μm.

Next, we assessed how GRFT affected EBOV and MARV entry, focusing on three fundamental steps (attachment, internalization, and membrane fusion). Virus-like particles (VLPs), consisting of EBOV or MARV proteins (Ebola and Marburg VLPs, respectively), were fluorescently labeled with a lipophilic dye (DiI) and observed using confocal laser scanning microscopy (Fig. 2A). At each entry step (i.e., 0, 2, and 5 h post-temperature shift [h.p.t.] assuming attachment, internalization, and fusion, respectively), we quantified the number of DiI-positive signals per cell, average size, and average total fluorescence intensity of individual DiI signals (Fig. 2B). At 1.5 h.p.t., the cells were treated with trypsin under conditions designed to remove cell surface-bound VLPs (Fig. S1). We found that the number of GRFT-treated VLPs was significantly lower than that of dimethyl sulfoxide (DMSO)-treated VLPs at all time points. Instead, we detected the enlarged DiI signals of GRFT-treated Ebola and Marburg VLPs at the attachment step (i.e., 0 h.p.t.) (Fig. 2A, white arrowheads). As correlated with this phenomenon, the average size and total fluorescence intensity of GRFT-treated VLPs were significantly higher than those of DMSO-treated VLPs at 0 h.p.t., whereas no enlarged signals were observed in the cells after the trypsin treatment (i.e., 2 and 5 h.p.t.) (Fig. 2A and B). On the other hand, these parameters (average size and average total fluorescence intensity) of GRFT-treated VLPs were almost similar to those of DMSO-treated VLPs when analyzed without enlarged signals (Fig. S2). Moreover, the number of GRFT-treated filovirus VLPs attached to the cell surface was significantly lower than that of DMSO-treated VLPs (Fig. S2). These data suggested that a large fraction of GRFT-treated VLPs might be aggregated during the initial incubation process. Electron microscopy further revealed that both Ebola and Marburg VLPs were indeed aggregated under the GRFT treatment (Fig. 2C). Since a reduction in the number of GRFT-treated VLPs was consistently observed in the cells throughout the time-course analysis (Fig. 2B; Fig. S1), GRFT-mediated VLP aggregation might have resulted in reduced numbers of individually countable, non-aggregated VLPs attached to the cell surface and subsequent internalization. Finally, we analyzed membrane fusion efficiency by assessing the enlargement and enhancement of DiI signals. We observed that the average size and average total fluorescence intensities of individual DiI signals similarly increased in both DMSO- and GRFT-treated filovirus VLPs at 5 h.p.t. (Fig. 2A; Fig. S2), indicating that GRFT had no effect on the membrane fusion step.

### GRFT neither induces aggregation of Lassa and Lujo VLPs nor inhibits their attachment to cell surfaces

**Fig 3:**
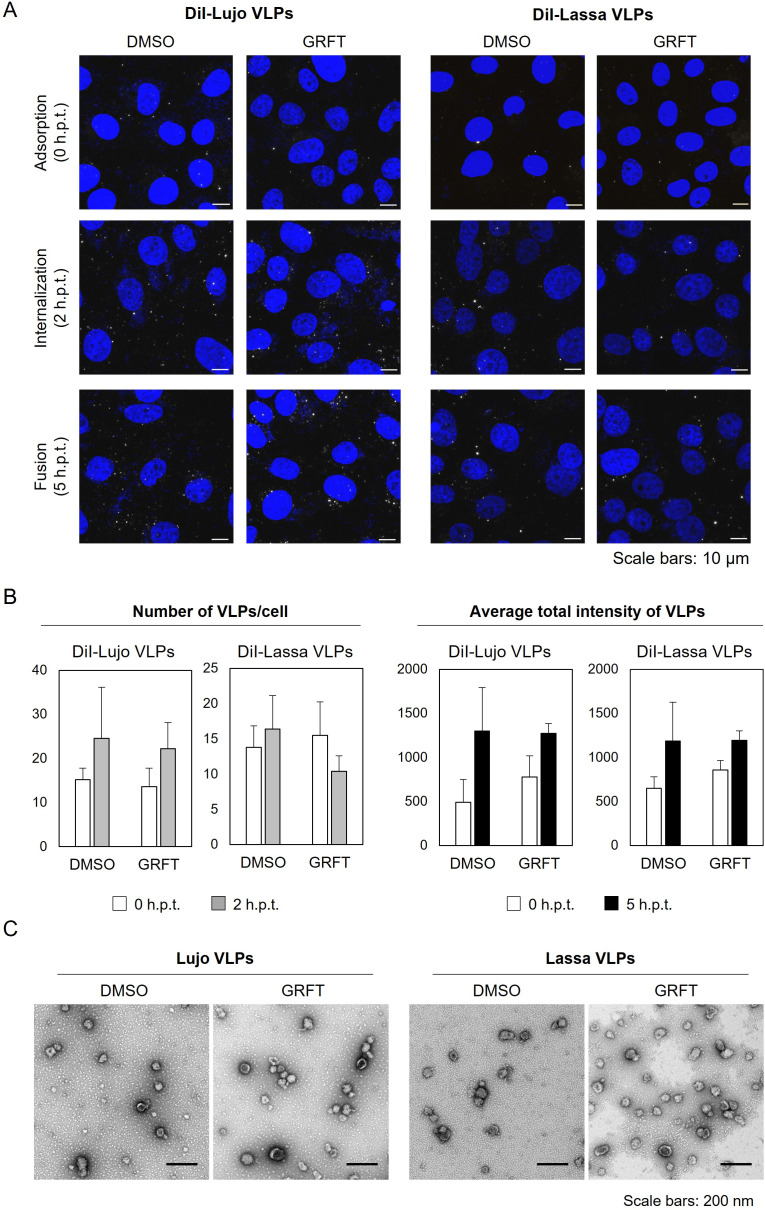
Fluorescent and ultrastructural observation of VLPs derived from arenaviruses. (**A and B**) Visualization of Lujo and Lassa VLPs. DMSO- or GRFT-treated DiI-labeled Lujo and Lassa VLPs were adsorbed into Vero E6 cells and incubated for 30 min at room temperature. After adsorption, the cells were incubated at 37°C. DiI signals on the cell surface and in the cytoplasm were monitored at 0, 2, and 5 h post-temperature shift (h.p.t.) using confocal laser scanning microscopy (**A**). The nuclei were stained with 1 μg/mL Hoechst 33342. Scale bars represent 10 μm. The numbers of DiI signals in the cells and average total fluorescence intensity of the DiI signals were quantified (**B**). The data represent the means ± standard deviations of three independent experiments. (**C**) Electron microscopy of Lujo and Lassa VLPs. Purified Lujo or Lassa VLPs were incubated with DMSO or GRFT for 30 min at 37°C, followed by negative staining. Scale bars: 0.2 μm.

We also assessed the effects of GRFT on the cellular entry of VLPs consisting of LASV and LUJV proteins (Lassa and Lujo VLPs, respectively). We found no significant differences in the numbers of VLPs attached (0 h.p.t.) and internalized per cell (2 h.p.t.) upon GRFT treatment when compared with their DMSO controls (Fig. 3A and B), whereas a positive control anti-LUJV GP neutralizing monoclonal antibody (LGN3-50-1) significantly reduced the number of internalized Lujo VLPs (Fig. S3). These observations suggested that GRFT had no significant effect on the attachment or internalization processes of Lujo and Lassa VLPs. Enhanced DiI signals were observed regardless of the treatment at 5 h.p.t., indicating successful membrane fusion induced by these VLPs; however, no significant difference in DiI signal intensity was detected between GRFT-treated and DMSO-treated VLPs (Fig. 3B). This did not necessarily indicate that GRFT had no effect on VLP-induced membrane fusion since a known fusion inhibitor, LHF-535, also failed to exhibit anti-fusion activity in this assay (Fig. S3), suggesting that the assay might not be reliable for assessing fusion inhibition in arenaviruses. Unlike Ebola and Marburg VLPs, particle aggregation was not notable in electron microscopy (Fig. 3C).

### N-glycosylation sites and surrounding amino acid residues are important for GRFT binding to LASV and LUJV GPs

**Fig 4:**
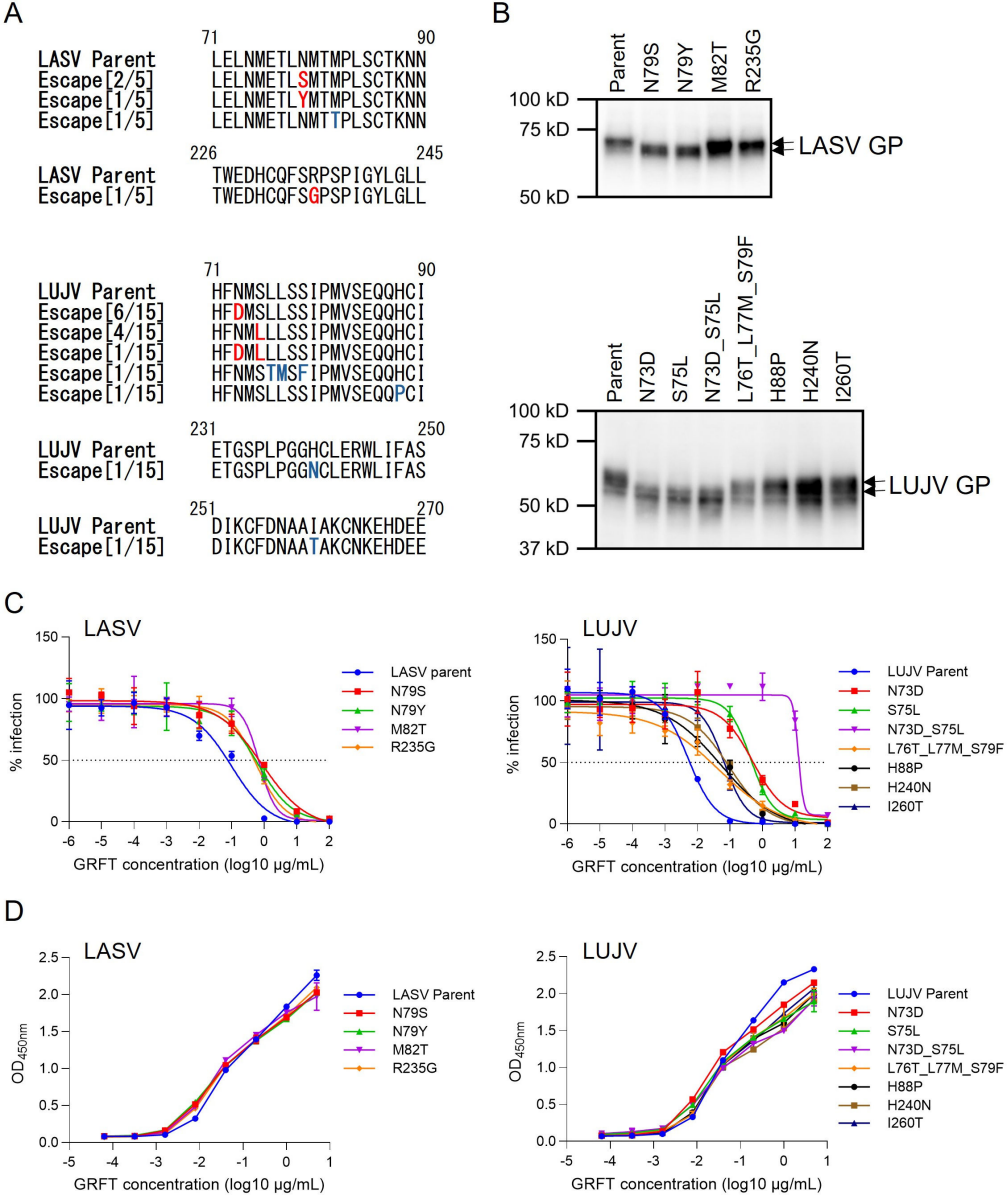
Amino acid substitutions found in escape mutant GPs of LASV and LUJV. Amino acid sequences in the indicated positions of LASV and LUJV GPs are shown (**A**). Amino acid substitutions found in the escape mutants selected in the presence of GRFT are shown in red (N-glycosylation motif) and blue (others). Molecular sizes of parent and mutant GPs were analyzed by Western blotting using lysates of HEK293T cells transfected with pCAGGS encoding each GP gene (**B**). Reduced susceptibility of each escape mutant was confirmed using VSIVs pseudotyped with parent and escape mutant GPs (**C**). Averages and standard deviations (triplicate) are shown. (**D**) Binding affinities of GRFT to wild-type (WT) and mutant GPs of LASV and LUJV were examined by the GP-GRFT binding ELISA. Data represent the means and standard errors of three independent experiments.

To identify amino acid residues important for the interaction with GRFT, we tried to select GP escape mutants using replication-competent rVSV-EBOV, rVSV-MARV, rVSV-LASV, and rVSV-LUJV. However, no rVSV-EBOV or rVSV-MARV mutant able to grow in the presence of GRFT was obtained. On the other hand, several escape mutants of rVSV-LASV and rVSV-LUJV were successfully selected, and amino acid substitutions in the GP genes were analyzed for comparison between each parent virus and its escape mutants (Fig. 4A). In the 5 LASV GP escape mutants obtained, amino acid substitutions were found at positions 79 (Asn-to-Ser [2/5] or Asn-to-Tyr [1/5]), 82 (Met-to-Thr), or 235 (Arg-to-Gly [1/5]). Of these, the substitution of Asn at position 79 was predicted to eliminate an N-linked sugar chain, since this position was a part of well-known potential N-glycosylation motif (Asn-X-Ser/Thr). In the 15 LUJV GP escape mutants, amino acid substitutions were found at positions 73 (Asn-to-Asp [6/15]), 75 (Ser-to-Leu [3/10]), 73 and 75 (Asn-to-Asp and Ser-to-Leu, respectively [1/15]), 76–77 and 79 (Leu-to-Thr, Leu-to-Met, and Ser-to-Phe, respectively [1/15]), 88 (His-to-Pro [1/15]), 240 (His-to-Asn [1/15]), or 260 (Ile-to-Thr [1/15]). Similar to the substitutions at position 79 in LASV GP, the substitutions of Asn-to-Asp and Ser-to-Leu at positions 73 and 75, respectively, in LUJV were predicted to eliminate an N-linked sugar chain at position 73, since these substitutions disrupted potential N-glycosylation sites. Reduced molecular sizes of mutant LASV and LUJV GPs that lost N-linked sugar chains were confirmed by Western blotting (Fig. 4B).

Then, the parent and mutant GP genes were cloned into the expression plasmids, and pseudotyped VSIVs were generated to test the inhibitory activity of GRFT. We confirmed that these amino acid substitutions affected the susceptibility to GRFT, whereas none of the substitutions conferred complete resistance to the lectin (Fig. 4C; Table S3). All the LASV GP mutants showed approximately 6–9 times higher IC_50_ values (0.49–0.70 µg/mL) than parent GP (0.076 µg/mL). Among the LUJV GP mutants, two glycosylation mutants, each with a single substitution (N73D or S75L), showed higher IC_50_ values (0.52 and 0.47 µg/mL) than the parent (0.0058 µg/mL) and the other mutants (0.045–0.077 µg/mL). It was noteworthy that double substitution (N73D and S75L) in LUJV GP resulted in a more significant reduction of the inhibitory activity of GRFT (IC_50_ = 13.30 µg/mL). Interestingly, the LASV and LUJV GP mutants exhibited GRFT binding levels equivalent to or only slightly lower than those of the respective wild-type GPs (Fig. 4D).

**Fig 5:**
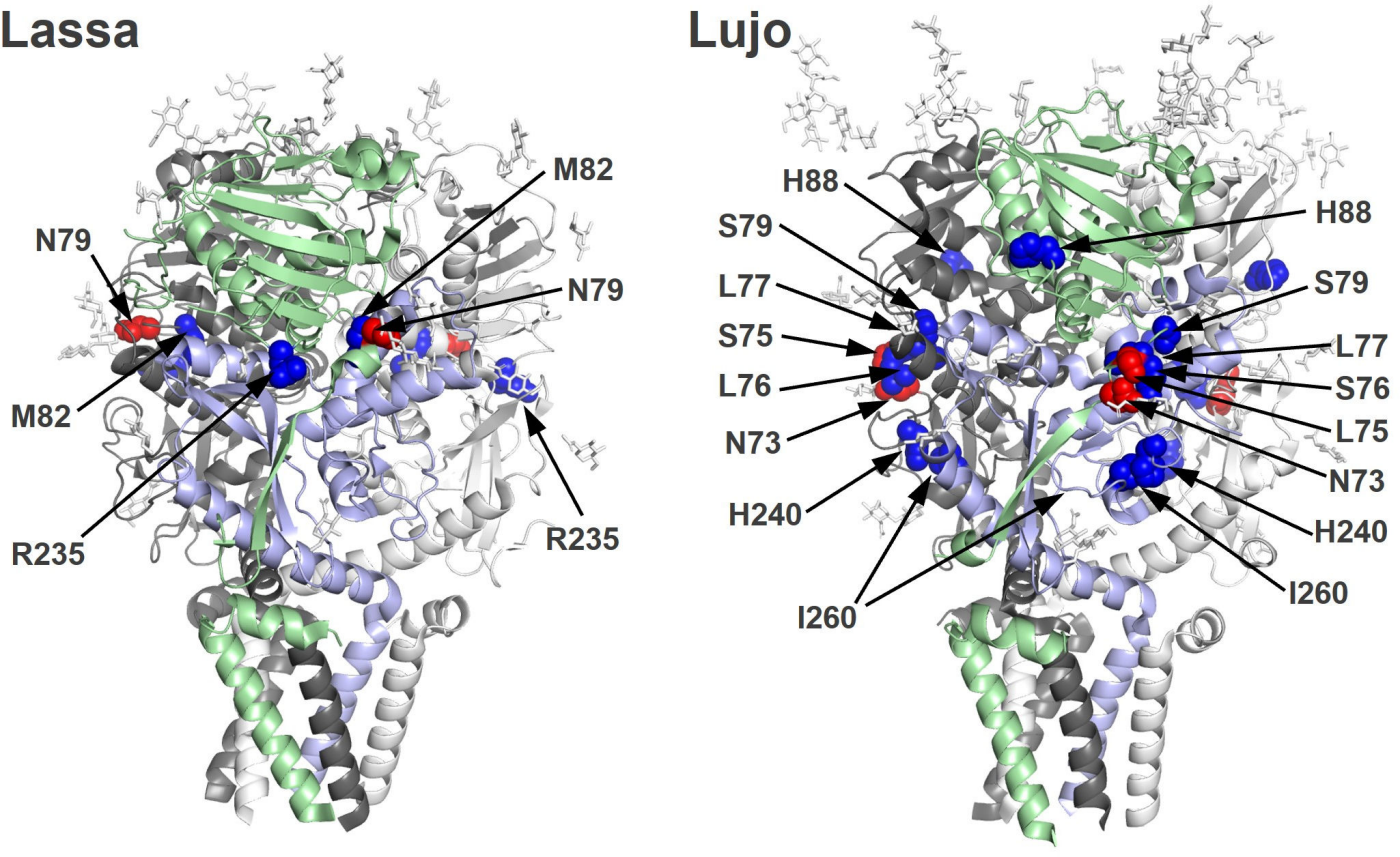
Substituted amino acid residues mapped on the LASV and LUJV GP trimeric structures. The amino acid residues critical for escape from the inhibitory activity of GRFT are mapped on the GP trimer structures of LASV and LUJV (PDB codes: 7PUY and 8P4T, respectively). The GP precursor proteins are processed by host proteases to yield a complex of a structured signal peptide (SSP), GP1, and GP2. The foreground molecules are colored in pale green (GP1 and SSP) and light blue (GP2). The locations of amino acid substitutions observed in the escape mutant GPs are shown as a spherical representation in red (N-glycosylation motif) or blue (others). Images were created using PyMOL (Schrödinger LLC, New York, NY, USA).

Amino acid positions corresponding to the substitutions found in the escape mutants were mapped on the 3D structures of LASV and LUJV GP trimers (Fig. 5). In LASV GP, Asn and Met at positions 79 and 82, respectively, present at the upper side surface of the GP1 subunit, were located in close proximity to Arg at position 235 within GP1. In LUJV GP, Asn, Ser, Leu, Leu, and Ser at positions 73, 75, 76, 77, and 79, respectively, formed a consecutive surface region on the upper side of GP1, and His at position 88 is located near these residues. Interestingly, His and Ile of the GP2 subunit at positions 240 and 260, respectively, were located close to the N-glycosylation site (i.e., Asn at position 73).

## DISCUSSION

Viral hemorrhagic fevers remain a major global health concern, with few effective treatments or preventive measures currently available (1), underscoring the urgent need for new and broadly effective antiviral strategies. A mannose-binding lectin, GRFT, has been studied as a potent broad-spectrum antiviral agent against a wide array of enveloped viruses, including EBOV (16, 18–24). Our study further demonstrated its broad cross-family antiviral activity against MARV (*Filoviridae*) and LASV/LUJV (*Arenaviridae*), as well as CCHFV (*Nairoviridae*). Variations in susceptibility to GRFT among the tested viruses are likely attributable to differences in the density and spatial arrangement of mannose residues, the extent of N-linked glycan processing, and the structural accessibility of high-mannose glycans on their envelope glycoproteins (29–32). The relatively lower susceptibility of EBOV and MARV to GRFT may also be explained by the structural characteristics of filovirus particles. Filoviruses exhibit a filamentous morphology with a substantially greater surface area than arenaviruses and nairoviruses, which likely results in a higher number of GP molecules per virion (33). In addition, filovirus GPs contain a glycan cap (GC) and mucin-like domain (MLD), both of which are densely decorated with N- and O-linked glycans (29, 30). Such features may increase the number of GRFT molecules required to inhibit filovirus entry.

Our analyses suggest that GRFT exerts its antiviral activity by inducing aggregation of EBOV and MARV particles. This inhibitory mechanism relies on virion aggregation through the bivalent nature of GRFT and is consistent with one of the proposed modes of HIV-1 inhibition by GRFT (34–36). In addition to this, cross-linking of surface glycoprotein molecules on HIV-1 virions has also been reported to contribute to the antiviral activity of GRFT (35). However, given the wide surface area of filamentous filovirus particles, such cross-linking effects on virions are likely less significant for EBOV and MARV than for HIV-1, since efficient inhibition of filoviruses would require a larger number of GRFT molecules. Although our confocal laser scanning microscopy did not provide clear evidence for the inhibitory activity of GRFT during VLP entry, some antiviral mechanisms of GRFT at this step cannot be ruled out, since the sensitivity and accuracy of the assay were likely compromised by VLP aggregation and because the analysis was performed using only a single cell line. One potential mechanism is that GRFT may alter the spatial arrangement or stability of GP trimers through its glycan-dependent interaction, thereby reducing GP binding to host receptors and/or conformational changes required for GP-mediated membrane fusion. Another possibility is that GRFT binding to GC or MLD may inhibit attachment to the surface of specific cells expressing C-type lectins, which compete with GRFT for glycan binding (37).

By contrast, electron microscopy observations provided no evidence of aggregation of Lassa and Lujo VLPs, suggesting that, unlike filoviruses, viral particle aggregation is not the primary mechanism of GRFT-mediated inhibition of LASV and LUJV entry into cells. Furthermore, our confocal laser scanning microscopy did not detect significant inhibitory activity of GRFT in VLP attachment to the cell surface, internalization, or membrane fusion. Nevertheless, the identification of escape mutations offers important insights into the inhibition mechanism. In LASV GP1, N79 and the neighboring residues M82 and R235 emerged as key sites involved in GRFT recognition. These positions are distant from the binding sites of the cellular receptors (α-dystroglycan and LAMP1), making direct interference with receptor engagement by GRFT unlikely (38, 39). N79 on LASV GP1 is a critical N-linked glycosylation site, heavily decorated with oligomannose-8/9 glycans (31), and plays an important role in shielding the GP2 fusion loop from immune recognition (40). GRFT targeting glycans at N79 may inhibit viral entry either by preventing the conformational change of GP or by cross-linking GP molecules on the viral particle. Although M82 is not directly involved in glycosylation, it may play a structural role in maintaining the prefusion conformation of the GP trimer (40), and thus, mutations at M82 could alter the structure of GP trimers, potentially reducing the GRFT recognition of the sugar chain at N79. Since M82 is a buried residue located immediately adjacent to the N79 glycan site, it is also possible that replacing this bulky hydrophobic methionine with a smaller, more polar threonine may influence the local packing in this region. This change may alter the orientation or dynamics of the N79 glycan and thereby reduce GRFT binding. In contrast, R235 is a fully exposed residue located adjacent to the N79 glycan. The R235G substitution replaces the positively charged, surface-exposed arginine with glycine, thereby altering the local electrostatic environment and surface topology. These changes may influence GRFT binding by both modifying the protein surface contacted by GRFT and by affecting the orientation or dynamics of the N79 glycan. Interestingly, R235 is part of a known neutralizing antibody epitope on GP1 (40). GRFT may recognize the structure formed by N-linked glycans at N79, together with nearby amino acid residues such as R235, and immobilize two monomers at the trimer core, thereby preventing the conformational change required for membrane fusion in a manner similar to that of some LASV-neutralizing antibodies targeting R235.

In LUJV GP, mutations at N-linked glycosylation site N73 and at surrounding amino acid residues were found to be critical for viral escape from the GRFT-mediated inhibition. While the glycosylation profile of LUJV GP remains less well defined than that of LASV GP, structural similarities between LASV and LUJV GPs suggest that N73 likely carries oligomannose-8/9 glycans, which serve as the primary target of GRFT, as well as N79 in LASV GP (41). Notably, multiple escape mutations that did not involve the loss of glycans were identified, and these were distributed across GP1 and GP2 subunits, suggesting a broader interaction interface on LUJV GP than on LASV GP. This may account for the higher antiviral potency of GRFT observed against LUJV. H88 is surface-exposed but is positioned somewhat distal to the N73 glycan. Replacing histidine with proline introduces restricted backbone flexibility at this position, which may alter the local surface conformation and, consequently, affect how the N73 glycan is presented to GRFT. I260 in LUJV GP2 is positioned internally but interacts with H240, which forms part of a loop located near the N73 glycan. Replacing isoleucine with threonine introduces a smaller, more polar side chain into this partially buried environment, which may alter the conformation or stability of this loop and, in turn, influence the local architecture of the GP1–GP2 interface. These changes could affect how the N73 glycan is oriented or supported at its base, thereby contributing to reduced GRFT binding. Thus, GRFT likely interacts with both N79 glycan and surrounding polypeptide structures of LUJV GP, locking its prefusion conformation and thereby inhibiting viral entry into cells. It is also conceivable that GRFT cross-links and immobilizes LUJV GP on the viral particle surface. However, it should be noted that all escape mutants of LASV and LUJV GPs remained sensitive to high concentrations of GRFT, suggesting the presence of multiple binding sites and additional mechanisms by which GRFT engages glycans other than LASV N79 and LUJV N73 on their GPs. Accordingly, only a limited difference was observed in the binding affinity of GRFT between wild-type and escape mutant GPs, suggesting that GRFT may recognize multiple N-linked glycosylation sites on GP.

Several other mannose-binding lectins, such as cyanovirin-N, scytovirin, and banana lectin, show notable antiviral activities against hemorrhagic fever viruses (16, 42–45). Compared with these lectins, GRFT demonstrates superior antiviral potency, translational progress, and manufacturability. GRFT elicits only minimal gene-expression changes in human cells and shows no detectable toxicity in mice (46, 47). In our cell viability assay, GRFT also did not show more than 50% cytotoxicity in any of the tested cell lines at concentrations up to 100 µg/mL (Fig. S4), and the selectivity index values for infectious LASV, LUJV, and CCHFV all exceeded 100 (Table S2), indicating a broad *in vitro* safety margin for GRFT. Q-GRFT, a recombinant oxidation-resistant variant of GRFT formulated as an intranasal spray, has already advanced to phase I clinical trials (48, 49). Moreover, recombinant GRFT can be produced at scale in plants, *E. coli*, and cell-free expression systems, supporting rapid deployment in outbreak settings (50–52). However, several challenges need to be addressed before GRFT can be advanced as a viable therapeutic option. For example, oral administration is unlikely to be practical due to its poor stability and bioavailability in the gastrointestinal tract, necessitating alternative delivery routes or formulation strategies to achieve effective systemic exposure (53). In addition, previous findings on its efficacy against EBOV suggest that further optimization, such as enhancing target specificity, extending *in vivo* half-life, or developing combination approaches, may be required to translate its potential into effective clinical application (24).

During outbreaks of viral hemorrhagic fevers, current strategies rely primarily on rapid diagnosis, supportive care, and stringent infection control measures to prevent further transmission. While these interventions are essential for mitigating the immediate impact of each outbreak, the development of effective countermeasures, including vaccines and antiviral therapies, remains a critical priority. GRFT represents a potent and versatile therapeutic agent against several hemorrhagic fever viruses. Once the GP-GRFT binding structures are clearly elucidated, it may become possible to rationally design modifications to GRFT, including increasing potency or avidity through multivalency or tandem constructs, as well as improving stability and manufacturability, such as through formulation and expression efficiency. A detailed understanding of its antiviral mechanisms will provide a strong foundation for further development, and continued research will be essential for advancing this promising natural product toward effective clinical interventions for viral hemorrhagic fevers.

## MATERIALS AND METHODS

### Preparation of recombinant GRFT and its mutants

Expression plasmids of recombinant GRFT and its D30A/D70A/D112A triple mutant were constructed by cloning synthesized DNA fragments, whose codons were optimized for *Escherichia coli*, into the NdeI/XhoI site of a modified pET28b plasmid, pDBHT-2, which has the N-terminus 6 × His tag gene adjacent to the cloning site (54). *Escherichia coli* strain BL21(DE3) harboring the expression plasmid of the desired proteins was cultivated in LB medium containing 25 mg/L of kanamycin at 37°C with shaking. When the OD_600_ reached 0.6, isopropyl-β-D-thiogalactopyranoside (IPTG) was added to the medium at a final concentration of 0.2 mM to induce the expression, and the culture was then incubated at 20°C for 20 h with shaking. Cells were harvested by centrifugation at 4,000 × *g* for 20 min. The collected cells were suspended in buffer A composed of 20 mM HEPES-NaOH (pH 8.0) and 200 mM NaCl and then disrupted with a UD-211 ultrasonic disruptor (TOMY SEIKO, Tokyo, Japan). After centrifugation at 40,000 × *g* for 60 min, the supernatant was loaded onto Ni Sepharose beads (GE Healthcare, Waukesha, WI, USA). After washing with a wash buffer composed of 20 mM HEPES-NaOH (pH 8.0), 200 mM NaCl, and 10 mM imidazole, the bound protein was eluted using a gradient of imidazole in buffer A. Fractions containing purified GRFT or the D30A/D70A/D112A mutant were collected and used for further experiments after dialysis against phosphate-buffered saline (PBS).

### Cells

Human embryonic kidney (HEK) 293T, African green monkey kidney Vero, Vero E6, and SW-13 cells were grown in Dulbecco’s modified Eagle’s medium (DMEM) (Sigma-Aldrich, St. Louis, MO, USA) supplemented with 10% fetal calf serum (FCS) (Sigma-Aldrich, St. Louis, MO, USA), 100 U/mL penicillin, and 0.1 mg/mL streptomycin (GIBCO, Waltham, MA, USA). Expi293F cells (kindly gifted by Dr. Kentaro Yoshii, Nagasaki University) were grown in Expi293 expression medium (Thermo Fisher Scientific, Waltham, MA, USA) and maintained at 37°C in 8% CO_2_.

### Viruses

Green fluorescent protein (GFP)-expressing, replication-incompetent VSIVs, whose infectivity was complemented with EBOV (Mayinga), MARV (Angola), LASV (Josiah), LUJV (200,809,232 Zambia), CCHFV (IbAr10200), and VSIV GPs (VSVΔG*EBOV, VSVΔG*MARV, VSVΔG*LASV, VSVΔG*LUJV, VSVΔG*CCHFV, and VSVΔG*VSIV), were generated as previously described (55). Infectious units (IUs) of these viruses were determined in Vero E6 cells using an IN Cell Analyzer 2500HS (GE Healthcare, Waukesha, WI, USA). Replication-competent VSIV carrying MARV, LASV, and LUJV GP genes instead of the VSIV G gene (rVSV-MARV, rVSV-LASV, and rVSV-LUJV) were generated as described previously, and virus titers were determined as plaque-forming units (PFU) (56, 57). Authentic EBOV (Mayinga), MARV (Angola), LUJV (200,809,232 Zambia), and VSIV were propagated in Vero E6 cells. LASV (LF2384) and CCHFV (IbAr10200) were propagated in Vero and SW-13 cells, respectively. Infectious titers of these viruses were also determined by plaque assays, according to previous studies, with slight modifications (58, 59). These viruses were stored at −80°C until use. All experiments with infectious EBOV, MARV, LASV, LUJV, and CCHFV were performed in biosafety level 4 (BSL-4) facilities in the Galveston National Laboratory (GNL) at the University of Texas Medical Branch at Galveston, in accordance with institutional guidelines. Relative percentages of infectivity were calculated by setting the number of infected cells in the GRFT-untreated control to 100%.

### Virus inhibition assays

Appropriately diluted VSVΔG*EBOV, VSVΔG*MARV, VSVΔG*LASV, VSVΔG*LUJV, VSVΔG*CCHFV, and VSVΔG*VSIV (2,000–5,000 IU/mL) in DMEM supplemented with 2% FCS (DMEM-2% FCS) were mixed with equal volumes of 10-fold serial dilutions of GRFT in DMEM-2% FCS, incubated at 37°C for 30 min, and inoculated onto Vero E6 cells in 96-well plates. The cells were incubated at 37°C for 18 h, and the numbers of GFP-expressing cells were counted using the IN Cell Analyzer. For EBOV, MARV, LASV, LUJV, CCHFV, and VSIV, appropriately diluted virus stocks (400–800 PFU/mL) were diluted with DMEM-2% FCS to yield approximately 50 PFU in 50 µL, mixed with equal volumes of 10-fold serial dilutions of GRFT in DMEM-2% FCS, incubated at 37°C for 30 min, and 100 µL of the mixture was inoculated onto Vero cells for LASV, Vero E6 cells for EBOV, MARV, LUJV, and VSIV, or SW-13 cells for CCHFV in 6-well plates. The cells were washed with DMEM-2% FBS and then overlaid with DMEM containing 0.6% Tragacanth (Thermo Fisher Scientific) for LASV, 1% Avicel RC-591 NF (IFF Pharmaceutical, New York City, NY, USA) for LUJV and CCHFV, or 0.8% Bacto Agar (Becton Dickinson and Company, Sparks, MD, USA) for VSIV. After incubation for 2 days (CCHFV), 5 days (LASV), 7 days (LUJV), 9 days (MARV), or 10 days (EBOV), the cells were fixed with 10% formalin and stained with 0.25% crystal violet.

### Purification and labeling of virus-like particles (VLPs)

VLPs consisting of GP, nucleoprotein (NP), and matrix protein (VP40) of EBOV and MARV were generated and purified as described previously (60–62). Equal amounts of the pCAGGS expression plasmids (5 µg each/75 × 10^6^ cells in a 125 mL Erlenmeyer flask) for EBOV or MARV VP40, GP, and NP were transfected into Expi293F cells using ExpiFectamine (Thermo Fisher Scientific), according to the manufacturer’s instructions. Forty-eight hours later, the culture supernatants were harvested and centrifuged at 440 × *g* for 5 min and then at 2,380 × *g* for 15 min to remove detached cells and cell debris, respectively. Then, VLPs were precipitated through a 30% sucrose cushion by ultracentrifugation at 20,700 × *g* for 1 h at 4°C with an SW32Ti rotor (Beckman, Fullerton, CA, USA). The precipitated VLPs were resuspended in TNE buffer (10 mM Tris-HCl [pH 8.0], 100 mM NaCl, 1 mM EDTA) and fractionated using a 30–60% sucrose gradient in TNE buffer at 132,900 × *g* for 2.5 h at 4°C with an SW32Ti rotor. The expression of EBOV and MARV VP40 in each fraction was confirmed by Western blotting using a mouse monoclonal antibody for EBOV (clone 3G5; IBT bioservices, MD, USA; 1:1000 dilution) and MARV (clone 1-17-1; 1:1000 dilution), respectively. The fractions corresponding to VLPs were collected, and VLPs were concentrated by ultracentrifugation at 20,700 × *g* for 1 h at 4°C with an SW32Ti rotor. The protein amount in the VLP suspension was determined using a Bradford protein assay kit (Bio-Rad Laboratories, CA, USA). The morphology of the purified VLPs was confirmed using transmission electron microscopy, and we found that the majority of VLPs exhibited a filamentous morphology. VLPs (100 µL of 1 µg/mL) were incubated with 6 μL of 10 μM 1,1'-dioctadecyl-3,3,3',3'-tetramethylindocarbocyanine perchlorate (DiI) (Thermo Fisher Scientific) in the dark for 1 h at room temperature with gentle agitation.

For the generation of LUJV and LASV VLPs, Expi293F cells were transfected with matrix protein (Z), GP, and NP expression plasmids of each virus (20 μg of Z and 15 μg each of GP and NP/150 × 10^6^ cells in a 250 mL Erlenmeyer flask) (63, 64). Culture supernatants were harvested using the same procedure as described above. For the initial pelleting down, VLPs in the supernatant were precipitated by centrifugation through a 20% sucrose cushion at 153,700 × *g* for 2 h at 4°C with an SW32Ti rotor, followed by fractionation through 10–50% iodixanol (Optiprep, Fresenius Kabi Norge AS, Oslo, Norway) at 186,700 × g with an SW32Ti rotor under the same conditions. The fractions containing VLPs were extracted and concentrated using Amicon Ultra Centrifugal Filters (Merck Millipore, Darmstadt, Germany). For DiI labeling, VLPs (97 μL of 125 µg/mL Lujo VLPs or 38 µg/mL Lassa VLPs) were incubated with 3 μL of 5 μM DiI in the dark for 1 h at room temperature with gentle agitation.

### Confocal laser scanning microscopy for imaging of internalization of DiI-VLPs

Visualization of the internalization of VLPs into living cells was performed as described previously (62). Briefly, DiI-labeled VLPs (0.04 µg/mL EBOV and MARV VLPs, 0.61 µg/mL Lujo VLPs, or 0.18 µg/mL Lassa VLPs) treated with 20 μg/mL GRFT or 0.5% DMSO for 1 h at room temperature were inoculated onto Vero E6 cells cultured in 35 mm glass-bottom culture dishes (MatTek Corporation, Ashland, MA, USA). The cells were incubated for 30 min at room temperature, followed by washing with the same medium. Then, the cells were incubated with 2 mL of phenol red-free DMEM containing 10% FCS at 37°C for 0, 2, and 5 h to analyze attachment, internalization, and membrane fusion, respectively. At 1.5 h.p.t, the cells were treated with 0.05% trypsin to remove the cell surface-bound VLPs. An anti-LUJV neutralizing monoclonal antibody (LGN3-50-1) and a small compound (LHF-535), thought to inhibit membrane fusion mediated by LASV (65) (HY-112762, MedChemExpress, Monmouth Junction, NJ, USA), were used as positive controls for Lujo and Lassa VLPs, respectively. To count the number of DiI-labeled VLPs per cell, the cells were fixed with 4% paraformaldehyde for 15 min at room temperature. Then nuclei were stained with 1 μg/mL of Hoechst 33342 (Cell Signaling Technology, Trask Lane, MA, USA) for 10 min at room temperature. Images were acquired using a confocal laser scanning microscope with an oil immersion objective lens at magnification 60 × (Fluoview FV3000, Evident Scientific, Tokyo, Japan) using FV31S-SW software (Evident Scientific). For measurement of the number of DiI-labeled VLPs, images of 4–20 sections were acquired in 0.5–1 µm steps. The number of DiI signals was determined in approximately 100 individual cells (approximately 5–10 dots/cell). The number, size, and total fluorescence intensity of DiI dots were analyzed using the ImarisCell module (Oxford Instruments, Oxfordshire, UK). In some experiments, aggregated VLPs (DiI signals with diameters greater than 3 µm) were excluded from the analysis.

### Electron microscopy

Transmission electron microscopy (TEM) was conducted as previously described (66). Briefly, purified EBOV (10 µg), MARV (10 µg), Lujo (2.25 µg), or Lassa (0.7 µg) VLPs were incubated with or without GRFT (20 μg/mL) for 30 min at 37°C and fixed with 2.5% glutaraldehyde (Electron Microscopy Sciences, Hatfield, PA, USA) in 0.1 M cacodylate buffer (pH 7.4) (Nacalai Tesque Inc., Kyoto, Japan) overnight at 4°C. Each sample was loaded onto a 200-mesh copper grid with a carbon-coated plastic film (Nisshin EM, Tokyo, Japan), immediately followed by glow discharge and negative staining with uranyl acetate solution (1%, wt/vol) for 15 s. The morphology of VLPs in each sample was observed using a JEM-1400 Flash microscope (JEOL, Tokyo, Japan) with an acceleration voltage of 80 kV.

### Selection of MARV, LASV, and LUJV GP escape mutants and sequence analysis

Selection of escape mutants was performed as described previously (67, 68). Briefly, 10-fold serial dilutions of rVSV-EBOV, rVSV-MARV, rVSV-LASV, and rVSV-LUJV were incubated with 4 μg/mL of GRFT for rVSV-LASV and rVSV-LUJV, or 20 μg/mL for rVSV-EBOV and rVSV-MARV, for 1 h at room temperature and inoculated into confluent Vero E6 cells grown in 6-well plates. After adsorption for 1 h, the inoculum was removed, and the cells were overlaid with Temin’s Modified Eagle Medium (MEM) (Thermo Fisher Scientific) containing 2.0% FCS, 1.0% Bacto Agar (Becton Dickinson and Company, Sparks, MD, USA), and 2–4 μg/mL of GRFT, and then incubated for 2–7 days at 37°C. Mutant viruses growing in the presence of GRFT were plaque-cloned and propagated in Vero E6 cells. Viral RNAs were extracted from the supernatant using a Viral RNA Mini Kit (QIAGEN, Hilden, Germany). The nucleotide sequences of the GP genes were determined by Sanger sequencing. Each GP gene was cloned into the pCAGGS plasmid and subjected to generation of pseudotyped viruses and GP expression in HEK293T cells. Western blotting of LASV and LUJV GPs was performed using a mouse anti-LASV GP2 antibody (LASGPC75-4-3), goat anti-mouse IgG polyclonal antibody conjugated with horseradish peroxidase (Thermo Fisher Scientific), and Immobilon Western (Millipore, Burlington, MA, USA).

### Cell viability assay

Vero, Vero E6, and SW-13 cells were seeded into 96-well plates and incubated overnight. The cells were then treated with 4-fold serial dilutions of GRFT starting at 100 µg/mL and incubated for 72 h prior to analysis. Cell viability was assessed using CellTiter-Glo (Promega, Madison, WI, USA) according to the manufacturer’s instructions. Relative luminescence units (RLUs) were measured using a GloMax 96 luminometer (Promega), and cell viability (%) was calculated by normalizing the RLUs of each sample to those of untreated control cells, which were defined as 100% viability. If cell viability did not fall below 50% at the highest concentration tested, the 50% cytotoxic concentration (CC₅₀) value was defined as >100 µg/mL. The selectivity index (SI) was calculated as the ratio of CC₅₀ to IC₅₀ (SI = CC₅₀/IC₅₀) for each cell line and virus tested. For samples in which the CC₅₀ exceeded the tested concentration range (> 100 µg/mL), SI values were estimated as greater than the ratio of 100 µg/mL to the corresponding IC₅₀ (i.e., SI > 100/IC₅₀).

### Expression and purification of recombinant LASV and LUJV GPs

Expi293F cells were transfected with pCAGGS plasmids encoding recombinant histidine-tagged soluble GP trimers from LASV and LUJV (69) using the ExpiFectamine (GIBCO, Waltham, MA, USA) according to the manufacturer’s instructions. At 120 h post-transfection, the culture supernatant was harvested and centrifuged at 800 × *g* for 10 min at 4°C to remove cellular debris. The recombinant GPs were purified from the clarified supernatant using a Ni–nitrilotriacetic acid affinity purification system (Invitrogen, Waltham, MA, USA) according to the manufacturer’s instructions. The purified recombinant LASV and LUJV GPs were used as coating antigens in the GP-GRFT binding ELISA.

### GP-GRFT binding ELISA

Binding of GRFT to LASV and LUJV GP was investigated as previously described (18–24). Briefly, ELISA plates (Thermo Fisher Scientific) were coated with purified recombinant LASV or LUJV GPs (50 µg/50 µL/well) diluted in PBS and incubated overnight at 4°C. Plates were washed with PBS containing 0.05% Tween-20 (PBST) and blocked with 1% bovine serum albumin (BSA) in PBST for 1 h at room temperature. After three washes with PBST, serial dilutions of purified GRFT prepared in PBST were added and incubated for 1 h at room temperature. After three washes with PBST, bound GRFT was detected by incubation with a 1:2,000 dilution of anti-GRFT rabbit polyclonal antibody (Thermo Fisher Scientific), followed by horseradish peroxidase (HRP)-conjugated goat anti-rabbit IgG secondary antibody (Invitrogen). After three additional washes with PBST, plates were developed using 3,3’,5,5’-tetramethylbenzidine (TMB) substrate (Sigma). The reaction was stopped with 1 N phosphoric acid, and absorbance was measured at 450 nm using an AO Microplate Reader (Azure Biosystems).

## Data Availability

The data that support the findings of this study are openly available in this article and are available from the corresponding author upon request.

## SUPPLEMENTAL MATERIAL

Supplemental figures jvi.00372-26-s0001.pdf Fig. S1 to S4.

Supplemental tables jvi.00372-26-s0002.pdf Tables S1 to S3.

ASM does not own the copyrights to Supplemental Material that may be linked to, or accessed through, an article. The authors have granted ASM a non-exclusive, world-wide license to publish the Supplemental Material files. Please contact the corresponding author directly for reuse.
