# Supplementary material for: Griffithsin-mediated inhibition of cellular entry of hemorrhagic fever viruses and insights into its mechanisms
Source: J Virol. 2026 Apr 27;100(5):e00372-26. doi: 10.1128/jvi.00372-26 (PMC13185625; doi:10.1128/jvi.00372-26)
Supplement: Supplemental figures — Fig. S1 to S4. [file jvi.00372-26-s0001.pdf]

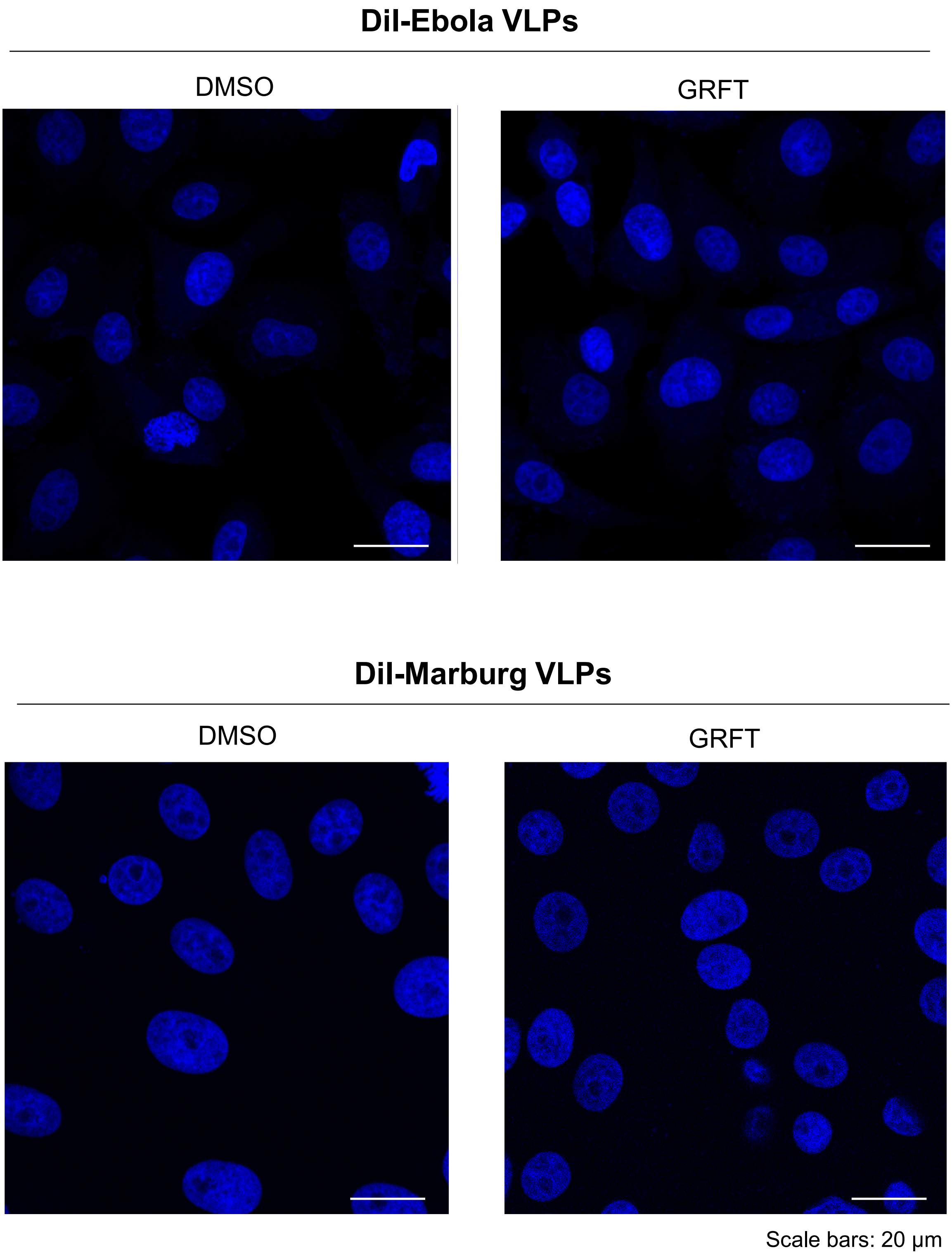

FIG S1. Detection of filovirus VLPs after trypsin treatment. DMSO- or GRFT-treated DiI-labeled EBOV and Marburg VLPs were adsorbed onto Vero E6 cells and incubated for 30 min at room temperature. Cells were then treated with 0.05% trypsin. Surface-associated DiI signals were analyzed by confocal laser scanning microscopy.

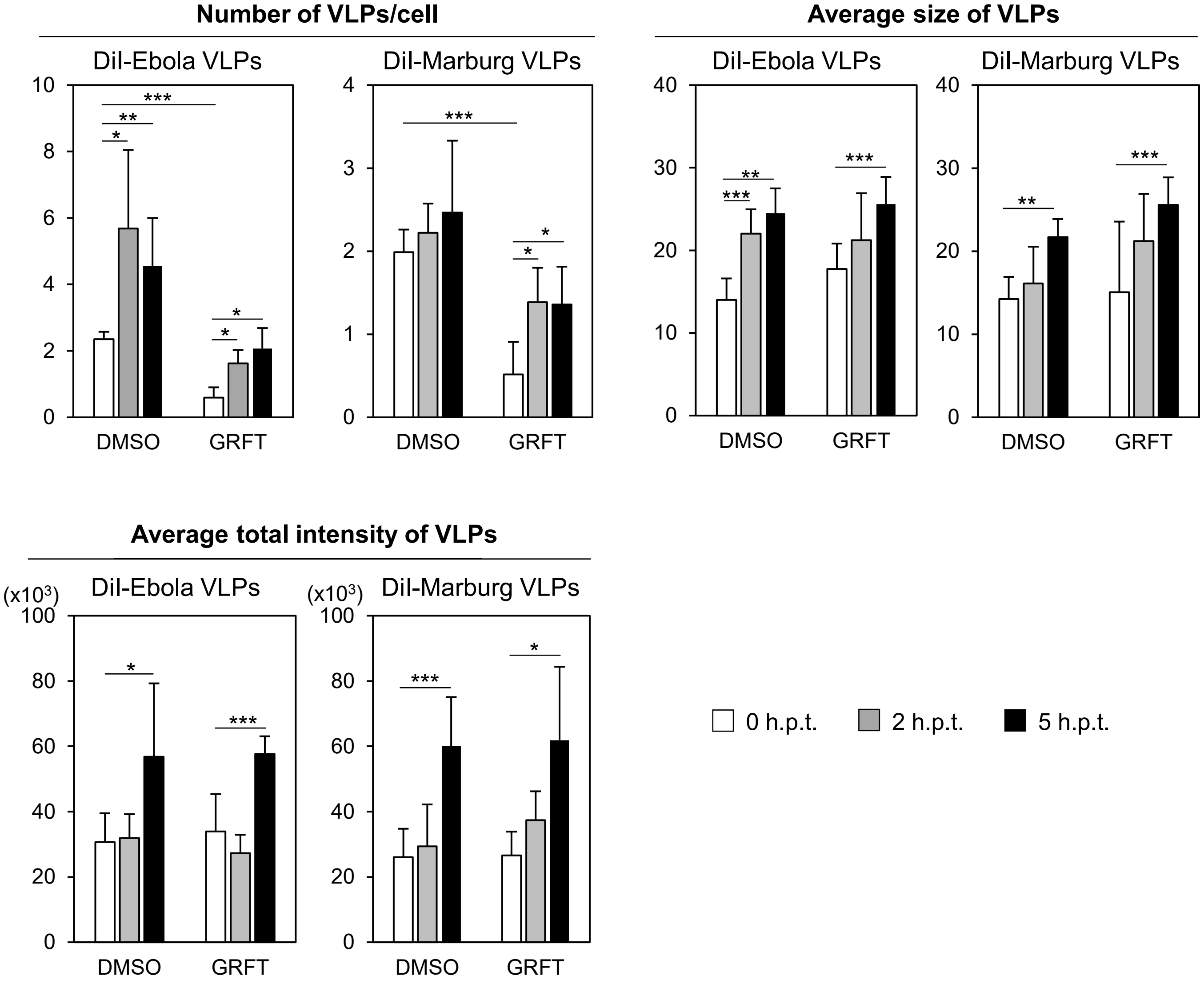

FIG S2. Quantification of GRFT-treated filovirus VLPs without enlarged DiI signals. DiI signals at the cell surface and in the cytoplasm were acquired at 0, 2, and 5 h post-treatment (h.p.t.). The number of intracellular DiI signals, mean particle size, and mean total fluorescence intensity were quantified using the ImarisCell module. Aggregated VLPs (DiI signals with diameters greater than 3 μm) were excluded from the analysis shown in Fig. 2B. Data represent the mean ± SD of three independent experiments. Statistical significance was determined by Student's *t*-test (**p* < 0.05, ***p* < 0.01, ****p* < 0.001).

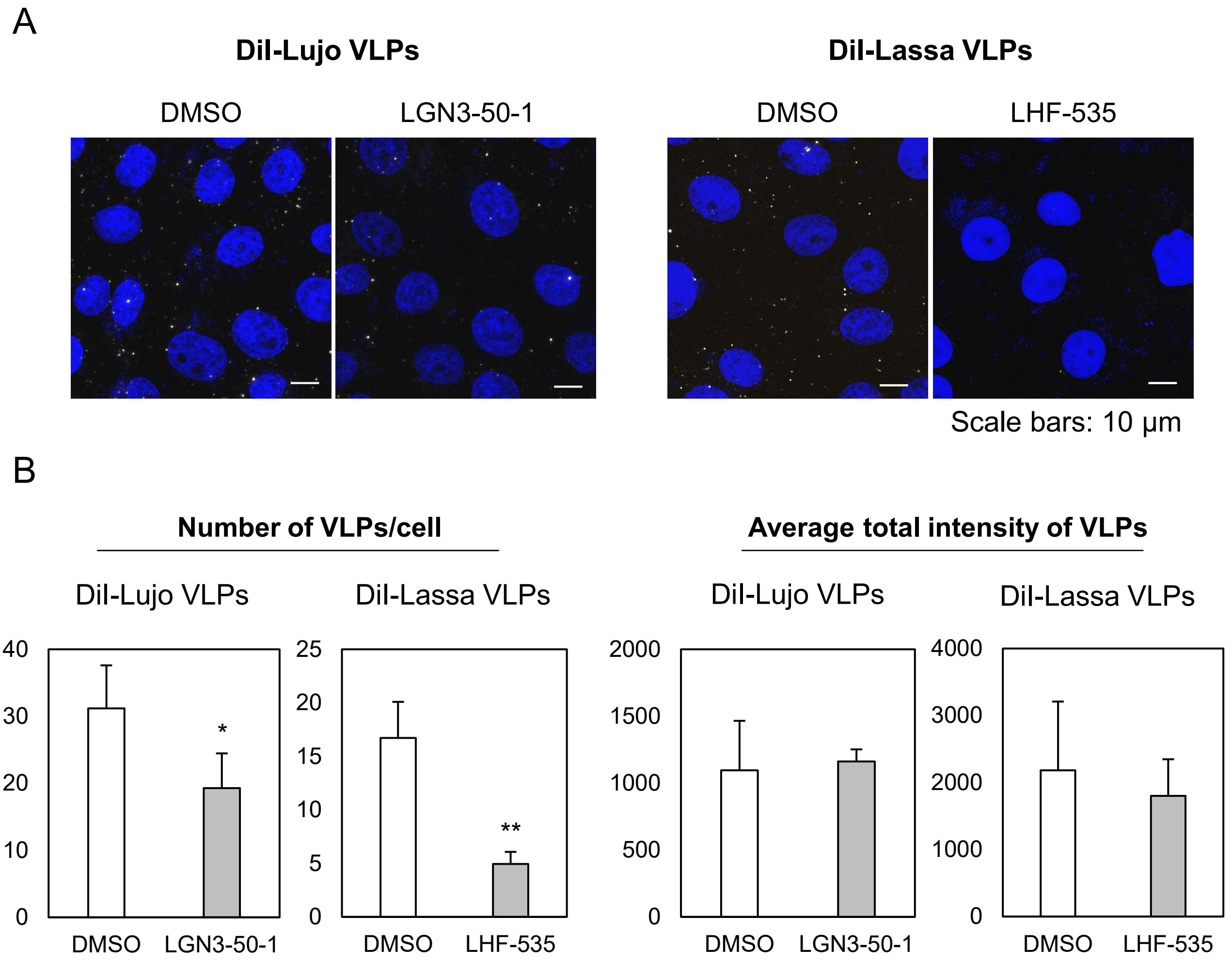

FIG S3. Validation of Lujo and Lassa VLP assays. (A-B) An anti-LUJV neutralizing antibody (LGN3-50-1) and a fusion inhibitor compound (LHF-535) were used for Lujo and Lassa VLPs, respectively. DiI-labeled Lujo and Lassa VLPs were treated with DMSO (0.5%), LGN3-50-1 (100 µg/ml) or LHF-535 (10 µM) , adsorbed onto Vero E6 cells, and incubated for 30 min at room temperature. After adsorption, the cells were incubated at 37° C. DiI signals on the cell surface and in the cytoplasm were monitored at 2 h post temperature shift using confocal laser scanning microscopy (A). Nuclei were stained with 1 µg/mL Hoechst 33342. Scale bars represent 10 µm. The numbers of DiI signals in the cells and total fluorescence intensity of the DiI signals were quantified (B). Data represent the means ± standard deviations of three independent experiments. Statistical analysis was performed using Student's *t*-test (**p* < 0.05, ***p* < 0.01).

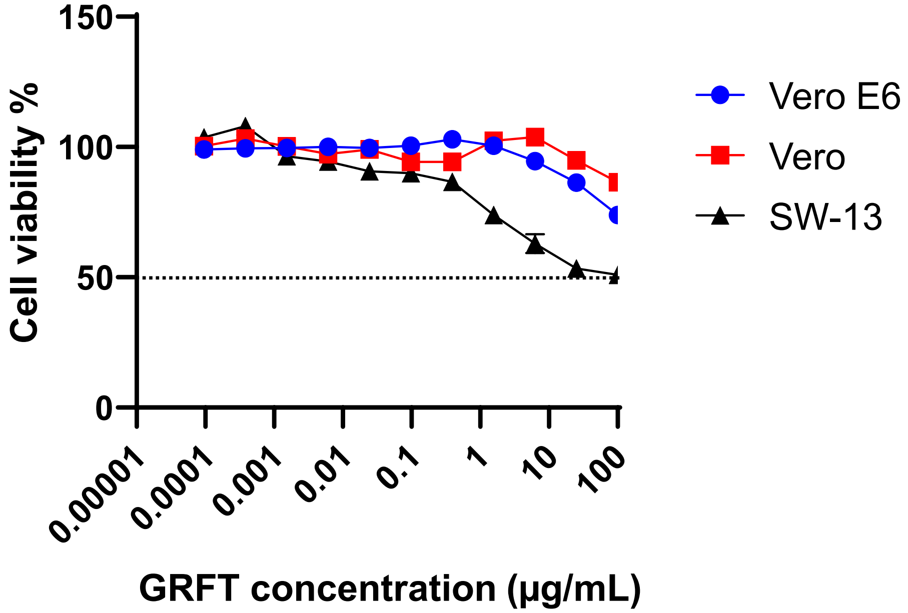

FIG S4. Cytotoxicity of GRFT in Vero E6, Vero, and SW-13 cells. Cells were treated with 4-fold serial dilutions of GRFT for 72 h, and cell viability was measured using the CellTiter-Glo assay. Cell viability (%) was normalized to that of untreated controls. Data represent the means and standard deviations of technical triplicates.
