# Supplementary material for: Griffithsin-mediated inhibition of cellular entry of hemorrhagic fever viruses and insights into its mechanisms
Source: J Virol. 2026 Apr 27;100(5):e00372-26. doi: 10.1128/jvi.00372-26 (PMC13185625; doi:10.1128/jvi.00372-26)
Supplement: Supplemental tables — Tables S1 to S3. [file jvi.00372-26-s0002.pdf]

Table S1. Inhibitory activity and selectivity index of GRFT against pseudotyped viruses.

Virus	IC_{50} (μg/mL)	CC_{50} (μg/mL)	SI
VSVΔG*EBOV	5.111	>100	>19.56
VSVΔG*MARV	0.7917	>100	>126.3
VSVΔG*LASV	0.04448	>100	>2248
VSVΔG*LUJV	0.003824	>100	>26149
VSVΔG*CCHFV	0.06705	>100	>1491
VSVΔG*VSIV	Undetermined	>100	Undetermined

Dose-response curves were generated using nonlinear four-parameter logistic regression based on the virus inhibition assay (Fig. 1A). The half-maximal inhibitory concentration (IC_{50}) for GRFT was calculated for each virus, representing the GRFT concentration (μg/mL) required to reduce virus infectivity by 50% based on the fitting curves. "Undetermined" indicates that a reliable IC_{50} could not be calculated due to poor curve fitting or failure to achieve 50% neutralization even at the highest concentration tested. The selectivity index (SI) was calculated as the ratio of 50% cytotoxic concentration (CC_{50}) to the IC_{50}. Because CC_{50} was not reached at concentrations up to 100 μg/mL (Fig. S2), CC_{50} was therefore recorded as >100 μg/mL, and SI values were reported as lower-bound estimates.

Table S2. Inhibitory activity and selectivity index of GRFT against infectious viruses.

Virus	IC_{50} (μg/mL)	CC_{50} (μg/mL)	SI
EBOV	120.6	>100	>0.8291
MARV	57.13	>100	>1.7503
LASV	0.8465	>100	>118.1
LUJV	0.3314	>100	>301.8
CCHFV	0.1624	>100	>615.9
VSIV	Undetermined	>100	Undetermined

Dose-response curves were generated using nonlinear four-parameter logistic regression based on the virus inhibition assay (Fig. 1C). The half-maximal inhibitory concentration (IC_{50}) for GRFT was calculated for each virus, representing the GRFT concentration (μg/mL) required to reduce virus infectivity by 50% based on the fitting curves. "Undetermined" indicates that a reliable IC_{50} could not be calculated due to poor curve fitting or failure to achieve 50% neutralization even at the highest concentration tested. The selectivity index (SI) was calculated as the ratio of 50% cytotoxic concentration (CC_{50}) to the IC_{50}. Because CC_{50} was not reached at concentrations up to 100 μg/mL (Fig. S2), CC_{50} was therefore recorded as >100 μg/mL, and SI values were reported as lower-bound estimates.

Table S3. Inhibitory activity of GRFT against VSVs pseudotyped with wildtype or escape mutant LASV or LUJV GPs

VSVΔG*LASV	IC_{50} (μg/mL)
Parent	0.07559
N79S	0.6992
N79Y	0.5485
M82T	0.6683
R235G	0.4935

VSVΔG*LUJV	IC_{50} (μg/mL)
Parent	0.005810
N73D	0.5189
S75L	0.4738
N73D_S75L	13.30
L76T_L77M_S79F	0.02643
H88P	0.04501
H240N	0.07677
I260T	0.06609

Dose-response curves were generated using nonlinear four-parameter logistic regression based on the virus inhibition assay (Fig. 4C). The half-maximal inhibitory concentration (IC_{50}) for GRFT was calculated for each virus, representing the GRFT concentration (μg/mL) required to reduce virus infectivity by 50% based on the fitting curves.
